# Living with Endometriosis: A Narrative Analysis of the Experiences of Kenyan Women

**DOI:** 10.3390/ijerph20054125

**Published:** 2023-02-25

**Authors:** Sadie Bergen, Doris Murimi, Caitlin Gruer, Gibson Munene, Atunga Nyachieo, Maureen Owiti, Marni Sommer

**Affiliations:** 1 Department of Sociomedical Sciences, Mailman School of Public Health, Columbia University, 722 W. 168th Street, New York, NY 10032, USA; 2 Endo Sisters East Africa Foundation, Laiboni Center, Off Lenana Rd., Nairobi P.O. Box 100798-00101, Kenya; 3 Institute of Primate Research, Karen, Nairobi P.O. Box 24481-00502, Kenya; 4 The Department of Obstetrics and Gynaecology, Kenyatta National Hospital, Nairobi P.O. Box 20723-00202, Kenya

**Keywords:** endometriosis, pelvic pain, menstrual disorders

## Abstract

Despite the high global prevalence of endometriosis, little is known about the experiences of women living with the disease in low- and middle-income contexts, including in Kenya and other countries across sub-Saharan Africa. This study captures the perspectives and recommendations of Kenyan women living with endometriosis through written narratives about the impact of the disease on their daily lives and their journeys through diagnosis and treatment. Thirty-seven women between the ages of 22 and 48 were recruited from an endometriosis support group in Nairobi and Kiambu, Kenya (February–March of 2022) in partnership with the Endo Sisters East Africa Foundation. Narrative data (written anonymous stories submitted through Qualtrics) were analyzed using a deductive thematic analysis methodology. Their stories revealed three themes related to their shared experiences with endometriosis: (1) stigma and disruption to quality of life, (2) barriers to acceptable healthcare, and (3) reliance on self-efficacy and social support to cope with the disease. These findings demonstrate a clear need for improved social awareness of endometriosis in Kenya and the establishment of clear, effective, and supportive pathways, with trained, geographically and financially accessible health care providers, for endometriosis diagnosis and treatment.

## 1. Introduction

It is estimated that one in ten women globally is living with endometriosis: a chronic inflammatory disease that causes pain and infertility [1,2]. The disease has no identified etiology, often takes years to diagnose, and has no cure [3]. Diagnostics remain limited, with confirmation of the disease often only ascertained surgically and management occurring through medication and surgery [3]. Although data remains limited, the symptomology often emerges during adolescence [4]. Studies conducted around the world found that between 25% and 100% of young people who undergo laparoscopy after reporting pelvic pain were diagnosed with endometriosis [5]. The average time from symptoms to diagnosis, even in high-income countries, is between four and eleven years [6]. This delay may be partly caused by the normalization of pain [7].

Despite the high global prevalence of the disease, the majority of qualitative and quantitative research on endometriosis has been conducted in a handful of high-income countries [8]. A limited body of research provides conflicting reports of the prevalence of this disease in countries across sub-Saharan Africa. Estimates range from a low of 0.2% in a sample of women in Northern Uganda [9] to a high of 48.1% among a sample of Nigerian women aged from 18 to 45 years [10]. A 2021 study in Nairobi, Kenya, found a prevalence of 8.9% among a sample of 224 women undergoing laparoscopic surgery [11]. Based on in-depth interviews with 16 women in Cape Town, South Africa, Roomaney, and Kagee [12], found that women used self-education, scheduling social and work events, and self-management techniques to cope with the physical, social, and psychological stressors of the disease. Another study in Cape Town focused on the impact of endometriosis on indicators of health-related quality of life among diagnosed women. Endometriosis negatively influences physical functioning, psychological well-being, relationships, and finances [13]. Additionally, a third study, also conducted in South Africa, examined fatigue as a secondary symptom of endometriosis in a sample of 25 women [14]. 

Other qualitative research, primarily from high-income countries, has examined the lived experiences of women with endometriosis across the domains of social and mental well-being. Evidence from across the globe is consistent in finding that endometriosis negatively affects the quality of life, including romantic and sexual relationships, social life, education, career, identity, and mental health [15,16,17,18]. Infertility associated with endometriosis has similarly negative effects on women’s mental health and long-term relationships [18,19]. Women have reported feeling that their pain was trivialized or dismissed by doctors and that medical providers lacked adequate information about the disease [18,20]. 

In Kenya, which is the site of this study, limited data exist on the prevalence of endometriosis [11]. However, two related topics to endometriosis—menstruation, and infertility—remain highly stigmatized and thus oftentimes are kept hidden [21,22]. For example, research conducted with school-aged girls in Western Kenya found that girls are often unprepared for menarche and hold beliefs that frame menstruation as secret or shameful [22,23]. This might pose challenges to their willingness to share menstruation-related concerns, including concerns about heavy bleeding that could be early signs of endometriosis.

Kenya has a population of 23.871 million women and girls, 65% of whom are aged 10–50 [24]: the most likely years of menstruation. The availability and quality of healthcare vary substantially by region, with the most specialized services available at hospitals in urban areas [25]. Given the diagnostic and support challenges identified with endometriosis, even in countries with improved healthcare access, there is an urgent need to better understand the needs that may exist for improved care and support among Kenyan women. 

The study described here was conducted in partnership with the Endo Sisters East Africa Foundation: a support and advocacy organization in Kenya. Given the sensitivity of conducting research with the vulnerable population of women living with endometriosis, a stigmatized disease, it was vital to partner with an organization that has deep knowledge of and the trust of this population. This partnership helped us recruit women to participate online. Due to the pandemic, we did not conduct in-person data collection. This study’s aim was to capture the lived experiences of women diagnosed with endometriosis in order to contribute to the minimal existing evidence on this issue in Kenya, including their pathways to diagnosis and the impact of endometriosis on women’s daily lives. 

## 2. Materials and Methods

We conducted a qualitative research study using a story-writing methodology with online anonymous data collection for women living in Nairobi and Kaimbu, Kenya, during the COVID pandemic (February–March 2022). The online approach was essential to assure researcher and participant safety when most of the population was home-bound. The story-writing methodology sought to capture the lived experiences of Kenyan adult women who were previously diagnosed with endometriosis. Participatory writing engages participants in a study with open-ended prompts to write personal stories or narratives that explore the meanings behind their behaviors and experiences [26,27]. An anonymous submission process is particularly useful when exploring topics that are in a given population [21,22]. Participants were drawn from the two counties in which the Endo East African Foundation hosts support groups, Nairobi and Kiambu.

### 2.1. Research Setting

Nairobi and Kiambu are Kenya’s largest counties, with populations of 4.3 million and 2.4 million [28]. These areas are inclusive of both urban and rural living experiences and have a range of availability in health care options. Kenya has a devolved governmental system, in which health policy is set by national authorities and services are managed and delivered at a county level [29], with the exception of national referral hospitals. Healthcare is provided through a combination of public, not-for-profit, and private health services. The availability and quality of healthcare varies substantially by region, and the most specialized services are available at hospitals in urban areas [30]. As of 2018, Nairobi had 724 health facilities across all levels of care, 20% of which were public. Nairobi also had 32% of Kenya’s doctors and eight referral hospitals. Kiambu had 487 health facilities, 22% of which were public, and had one referral hospital [31]. 

### 2.2. Sampling and Recruitment 

We used purposive sampling to select women (*n* = 37, ages 22–48) for inclusion in the study (Table 1), seeking to capture those who had previously been diagnosed with and were living with the disease. Participants were recruited through electronic communications (in order to follow safety precautions during the pandemic) from support groups hosted by EndoSisters East Africa. Fliers explaining the study and inviting participation were sent via WhatsApp and other e-communications. 

### 2.3. Data Collection

Participants were sent a link to a Qualtrics open-response survey with an informed consent form, basic demographic questions, and writing prompts. A four-week window of time was provided during which submissions could be submitted. Women were given the option of responding to one of two writing prompts: (1) What was your journey in seeking treatment and care for your endometriosis? or (2) Tell a story about an experience when endometriosis impacted your daily life (for example, social life, education, work). For both prompts, participants were asked to provide details on what happened, how they felt, how they managed, and advice they would give to other women who may be struggling with similar symptoms. 

Ethical approval was obtained from Columbia University in New York, USA, and from Kenyatta National Hospital-University of Nairobi in Nairobi, Kenya. 

## 3. Results

Three major themes were identified: (1) Stigma and disruptions to the quality of life, (2) barriers to acceptable healthcare, and (3) reliance on self-efficacy and social support to cope. The findings were not found to vary based on the location of the submission, with similar experiences reported from both study sites. 

### 3.1. Stigma and Disruptions to Quality of Life

The majority of participants across both sites described their endometriosis symptoms as debilitating. In stories about how endometriosis shaped their daily lives, multiple women used words such as “unbearable” and “excruciating” to characterize their symptoms, which included deep abdominal pain, nausea, vomiting, and heavy bleeding. These symptoms disrupted their quality of life across multiple domains of well-being, including education, professional lives, social and romantic relationships, and mental health. 

Many women first experienced their endometriosis symptoms as adolescents at school. These formative experiences were fresh memories for the adult participants. Women described suffering from agonizing and inexplicable pain and heavy bleeding during their school days, which caused them to miss class and had a negative impact on their academic performance. A number of women recalled instances where they performed poorly on tests or were unable to complete their assignments because of abdominal pain. Several described their frequent visits to the school nurse and local doctors’ offices during the school day. As one woman recalled:

*One time in primary school I had too many cramps that I started rolling on the floor feeling very powerless. I could not even speak. I was rushed to the dispensary and got an injection, but it made everything more painful. Teachers and doctors were so afraid I would die in their hands and my parents had to be called to pick me up as soon as they could…. I missed school for two more days and was behind in my studies.*

(Story 37, aged 27)

Important to note is this woman’s reference to ‘primary school’ as a difficult time for managing her period, which indicates the importance of raising awareness among young people, their caregivers, and teachers. 

The impact of endometriosis on educational experiences was also described as traveling beyond class performance. Some women shared the ways in which the school administration and the social environment compounded their negative experiences of the disease. In several cases, women remembered school as a site of formative stigmatizing experiences related to their symptoms. As one woman described:

*Our school matron would give me some painkillers, but they never worked. I would barely move and just wish to die. I would question God why I had to go through so much pain, yet my fellows would just act normal. I experienced low self-esteem as my school principal would even announce during assembly that I was seeking attention and threatened to suspend me from the school if I didn’t change. I used to miss classes and other activities.*

(Story 24, aged 26)

This description exemplifies one pathway through which stigmatizing experiences for adolescents with endometriosis compound existing patterns of gendered educational inequality. Women’s responses suggest that not only do school faculties and medical professionals not provide adequate practical and emotional support for their symptoms, but they may also exacerbate girls’ challenges by assigning stigmatizing labels such as “attention seeking” that discourage adolescents from seeking help for concerning menstrual symptoms. 

Women’s professional lives were also disrupted by their endometriosis experiences, with many struggling with incapacitating symptoms while in the workplace. More than one participant wrote about losing consciousness at work as she tried to “*persevere and push through*” the pain (Story 23). Another woman who passed out in the workplace reported feeling embarrassed and exposed after the experience, while others described missed days of work and critical reactions from colleagues and employers. A 33-year-old woman described being refused accommodations in a public event related to her work and being forced to suffer through an extended work proceeding before driving to the closest hospital. As she reflected, “*everywhere hurt and especially the emotional torture*” (Story 29). Others explained that their symptoms, combined with the negative reactions of employers, led them to quit jobs or change career paths. These experiences ranged from being pressured to share their entire medical history with an employer to justify their workplace engagement to the decision to seek employment online. As one woman shared:

*Because of my on -off working schedule and the criticism from others, also the guilt I feel for being so unreliable, I decided to stop going to work altogether. Been trying to do some online jobs and I’m happy since I’m able to pace myself and when I don’t feel well, I can rest without feeling guilty and resume when I feel up to it.*

(Story 26, aged 28)

Although the outcome was positive for this woman, online or remote work is limited to certain professional sectors and may be difficult to find. If women with endometriosis can only obtain reasonable workplace accommodations through online or remote professions, they may face negative economic outcomes. As with education, women described the many ways that endometriosis compounded existing gendered economic and professional inequalities. 

Additionally, compromising their quality of life, many women explained how as adults, lengthy periods and heavy bleeding prevented them from participating fully in their social and romantic lives. As one woman explained: I had to plan social events around my period, especially because I would now be on my period for at least 14 days. (Story 5, aged 38) Although this woman was primarily sharing her concerns in relation to her social life, the quote raises both physiological concerns, such as the potential risk of anemia from lengthy menstrual periods, and worries about the mental health impact of constrained engagement with others. 

A number of women shared more specifically how endometriosis disrupted their intimate relationships and caused insecurity and discomfort around sexual relationships. As one woman described:

*My friends started discriminating [against] me because I couldn’t hang out with them when I was on my period…I became self-conscious and feared sex because of fear of starting bleeding during the act. I faced rejection every time I raised my period issues to a potential lover.*

(Story 14, aged 25)

This example suggests that women experienced multiple forms of stigma because of their long-term endometriosis symptoms, including social exclusion and fear of romantic rejection. The ways in which their social interactions and physical abilities were affected contributed to their endometriosis, taking a toll on their mental health. This included profound feelings of hopelessness and even suicidality. On top of the drain of medical bills and isolation, one participant described ending up in a “*dark place where I just wanted to end it all*” (Story 5, aged 38). The same woman (above quote) who described facing rejection in her romantic relationships described a range of adverse mental health effects:

*In 2020 my bleeding advanced and made a permanent residency in me, I got tired and stopped seeking help or going anywhere...Endometriosis can make you go crazy, it opens doors for other illnesses and depression and anxiety disorders become inevitable. Endometriosis has made me hate being a woman!*

(Story 14, aged 25)

While these responses highlight the connection between severe endometriosis symptoms and quality of life, women’s descriptions of discrimination across multiple domains of their lived experience also suggest an absence of widespread knowledge about endometriosis. The lack of societal awareness and ongoing stigma around the disease compromised their psychosocial well-being and medical care and deeply limited engagement in their social and professional lives. 

### 3.2. Barriers to Acceptable Care

In writing about their journeys to endometriosis care, women described multiple barriers to receiving a formal diagnosis and finding acceptable treatment that mitigated at least some of their symptoms. Many women described how their pain was dismissed by those around them as “normal” menstrual cramping, which they felt invalidated their experiences and thus intensified their mental suffering. As one woman explained, *“[I] started noticing symptoms at the age of 17 years though this was not taken seriously as it was dismissed as a normal woman thing and that I was exaggerating*.” (Story 3, aged 44)

For some women, the normalization of menstrual pain by friends and family served as a barrier to seeking medical care. For others, the normalization of pain posed challenges once they had entered the medical system. Women described having their experiences dismissed by medical professionals, and further, that their symptoms were psychological: “*I kept being told that I was pretending and that it’s all in my head*” (Story 5, aged 38). These comments from people in positions of medical authority were articulated by the women as having set them back in their journeys to diagnoses by discouraging them from seeking further care. 

All of the women in the study had ultimately obtained endometriosis diagnoses, yet numerous participants described facing multiple challenges before receiving a formal diagnosis within the Kenyan health system. Many women felt that their doctors were not knowledgeable about endometriosis, with one woman articulating: “*Most doctors around Kenya are green about this situation*” (Story 13, aged 32). Others described feeling frustrated by the lengthy experience of visiting many different doctors and receiving multiple misdiagnoses before finding the “right” doctor and a proper diagnosis:

*It took me almost 7–8 years to finally have the problem at hand [identified] and several visits to different gynecologists.*

(Story 33, aged 22)

*Initially I [was] diagnosed with malaria, typhoid, ulcers until roughly 7 years later when I got proper medication and got [the] right diagnosis.*

(Story 20, aged 36)

Beyond a lack of knowledge, diagnostic delays were exacerbated by myths and misinformation shared by doctors. Multiple women wrote that they were advised to “*have a baby*” in order to resolve their symptoms, including the suggestion that it would reduce their pain, while another respondent was told that she could not have endometriosis because “*endo is for the affluent*” (Story 16, aged 30). This advice reflects how existing sociocultural beliefs and misperceptions around endometriosis may be negatively influencing the delivery of care. 

In addition to experiences with medical professionals, participants described societal structural barriers to receiving acceptable health care. Several women emphasized the high cost of medical care for those without adequate insurance:

*My medical journey officially started in 2016 when I was 24 and saw a gynecologist for the first time in my life…This was when I had a medical insurance, and I could afford a consultant.*

(Story 9, aged 30)

Other women highlighted geographic challenges in their journeys to a diagnosis. These ranged from struggling to access adequate care in more rural areas of Kenya to those who could afford to travel to Europe to find a doctor who could diagnose and treat their disease. Health system-related barriers such as these are particularly problematic for conditions such as endometriosis, given the multiple medical visits needed to diagnose the disease and the chronic nature of treating the symptoms. 

### 3.3. Coping with Self-Efficacy and Social Support 

Given the challenges of accessing medical care and support throughout their endometriosis journey, women ultimately described learning to rely on themselves and those around them who believed in their symptoms. When asked to reflect on what advice they would offer to adolescent girls or women who were earlier along in their endometriosis journeys, many emphasized the importance of self-reliance. More specifically, participants wrote about the need to gain a sense of self-efficacy, or the belief in one’s ability to achieve a desired outcome, through self-knowledge of their own bodies and independent research about their symptoms. This, they felt, would allow them to advocate for themselves in medical encounters, including making sure that the wrong care was not received, a stressful reality described by some participants. As one woman articulated, the ability to speak up was required in managing this disease to avoid circumstances when medical care could otherwise go awry. As she emphasized:

*My advice to all women going through this [treatment], kindly search and search for a right solution, let no one rush you to theatre rooms to remove organs for you, period pains are real and there is no shame in going through it, but this pain needs medical attention and needs a voice so that we get and demand for local solution within Kenya.*

(Story 10, aged 32)

Along with highlighting the importance of self-advocacy and determination, such advice suggests the limited quality of existing healthcare options for endometriosis, including well-trained providers, for Kenyan women. 

In addition to self-reliance, the women emphasized the importance of social support from friends, family, and partners in coping with the mental and physical side effects of endometriosis. They shared that although many in Kenyan society may not understand the disease, it is still possible to be supported on the painful and oftentimes lonely journey. As one woman explained:

*I have amazing friends and family who have stood by me through it all. They waited for me outside the theatre for those long hours, they have listened to me vent and cry about a problem they don’t fully understand, and they have shown me all the things in the world I have to look forward to.*

(Story 17, aged 37)

Peer support was similarly described as critical for the women in emotionally and physically managing and surviving their journeys. All of the women participated in endometriosis support groups, with many emphasizing how these groups were essential in providing concrete information about a poorly understood disease, along with much-needed affirmation of the validity of their experiences. One woman articulated this sentiment:

*It’s very important to have a support focus group. That way you won’t feel alone, your struggles would be acknowledged by other women who believe you because they are also facing them, you will be encouraged by other women’s experiences.*

(Story 14, aged 25)

The existence of social support on their journeys appears to have increased self-efficacy for many of the women by bolstering their confidence to seek out and pursue treatment options that worked best for them. This helped them to feel more in control of how they managed their chronic disease rather than following a single, narrowly prescribed course of treatment. Finally, women were very clear about the importance of receiving an endometriosis diagnosis as early as possible. Their recommendation was for adolescent girls and women who have any concerning menstrual symptoms to seek out medical treatment “*until they are feeling at peace*” (Story 2, aged 34), without allowing the medical system to discourage or dismiss them. 

## 4. Discussion

In this qualitative study, Kenyan women wrote narratives describing their experiences with endometriosis, including their journeys to diagnosis and treatment and the impact of their symptoms on their daily lives. Their stories highlighted the debilitating impact of endometriosis and related stigma on their quality of life, existing barriers to adequate and acceptable medical care, and the essential need for self-efficacy and social support to cope with the disease. These findings are consistent with evidence across multiple global contexts that suggest women share similar lived experiences of endometriosis in spite of economic, social, and cultural differences [15,16,17,18]. Research in South Africa has similarly shown how this chronic illness negatively impacts multiple aspects of women’s well-being, including physical functioning, mental health, finances, and interpersonal relationships [32]. The findings from this study contribute important perspectives directly from Kenyan women, helping to build the limited evidence base on endometriosis across sub-Saharan Africa.

Debilitating endometriosis symptoms adversely affect the quality of life of the women who participated in the study through disruptions to their educational, professional, social, and romantic lives and negatively impact their mental health. Women described how overwhelming pain and other disruptive symptoms resulted in missed school and work and, in turn, compromised their potential educational and professional trajectories: findings that have been found in studies conducted in New Zealand, Puerto Rico, the United Kingdom, and elsewhere [17,33,34,35]. Kenyan women’s quality of life was further diminished by ongoing stigma and shame, which served to isolate those with endometriosis; participants shared how symptoms could lead to discrimination among their social groups and feelings of shame and fear in romantic relationships. These experiences suggest that women with endometriosis experience stigma that is at a level potentially more severe than existing studies of school-aged girls in Kenya which explored menstrual stigma [22,23]. These findings reinforce the call from Sims et al. [36] for more dedicated research into an endometriosis-related stigma.

Our findings also suggest a profound lack of societal awareness and understanding of endometriosis in Kenya. This is consistent with the outcome of a study on the prevalence of pain symptoms suggestive of endometriosis among 313 adolescents in Kenya, wherein 29% reported severe dysmenorrhea, but 94% had not heard of endometriosis [37]. To combat the limited knowledge about endometriosis, menstrual disorders education could be included within the Kenyan health education curriculum for relevant ages. In New Zealand, endometriosis education was in recent years incorporated into the health education curriculum in some schools with demonstrated improvements in adolescents’ awareness of the disease [38]. The onset of endometriosis may occur during adolescence, and if undiagnosed and subsequently not treated, symptoms may progress beyond the pain to infertility [5]. A related challenge, many of the women in our study noted, is the “normalization” of menstrual pain among those who they encountered from adolescence through adulthood. The frequent dismissal of their pain occurred from family, friends, teachers, school health professionals, and physicians. This is similar to findings from qualitative research conducted on endometriosis in Australia, the United Kingdom, Austria, Germany, and Hungary, which also indicated challenges throughout the life course of endometriosis-related pain being normalized [7,16,39,40,41,42]. 

Limited knowledge about endometriosis, the normalization of pain, and stigma were found to synergistically impact negatively on Kenyan women’s ability to be diagnosed and treated, similar to the kinds of diagnostic delays that are associated with endometriosis in many other contexts. A global, ten-country study found that women waited an average of 6.7 years to be diagnosed with endometriosis, with longer delays in countries with government-funded health care [17]. The Kenyan health system was also reported to be problematic, with participants describing visiting multiple physicians, being repeatedly misdiagnosed, and receiving inaccurate information about the disease. The latter challenges are consistent with research on women’s health-care-seeking experiences of endometriosis in the United States, the United Kingdom, Australia, and Brazil [43]. Such diagnostic delays suggest the need for more patient-centered care, including improved communication with patients about the lengthy process of elimination through which endometriosis is diagnosed. In Kenya, the availability and quality of healthcare vary substantially by region, with the most specialized services available at hospitals in urban areas [30,44]. This can pose challenges for those with symptomology who are living in rural areas. The diagnosis of endometriosis also requires laparoscopic surgery, which is not routinely available in most healthcare settings in Kenya because of the high upfront cost of medical equipment [45]. 

Overall, a lack of adequate numbers of qualified clinicians adds to the emotional trauma and financial distress of patients seeking relief as they seek out answers, oftentimes from primary care clinicians. One solution would be to increase access to gynecologists, who receive some training on endometriosis in medical school and residencies [46] so as to make expertise more available to those in need. As the women shared, inadequate provider training in Kenya extended to include the perpetuation of misinformation about how to best “treat” endometriosis. This was exemplified by multiple women who described having been advised by doctors to become pregnant to relieve their symptoms. Women in Hungary, Australia, and the United Kingdom have similarly reported being pressured to have a baby by clinicians [16,18]. Given that there are known associations between endometriosis and infertility, an issue; that is, particularly sensitive in a society where infertility is stigmatized [21], such clinical messaging may augment rather than relieve a woman’s situation. In a study of 75 Kenyan women with fertility problems (mean age 34), 94.4% reported that “as long as they could remember, they wanted to become a mother”, and 74.3% reported experiencing stigma because they did not have children or experienced fertility problems [21]. 

Many women wrote about the mental health toll of seeking a diagnosis and living with endometriosis, including language suggesting that the women had been living with significant emotional distress, including thoughts of suicide. A recent survey-based study conducted in South Africa found that in a sample of 202 adult women diagnosed with endometriosis, over 40% reported moderate to severe symptoms of depression [32]. In another study, Roomaney and Kagee noted that participants thought of endometriosis as an individual affliction and, therefore, did not seek social or emotional support [13]. These findings align with prior research on the psychological impact of endometriosis. Based on studies conducted in Australia, the United States, the United Kingdom, and Canada, a 2013 review of qualitative and quantitative evidence called emotional distress a “key feature of living with endometriosis” [43]. From a health system perspective, our findings highlight the need for a more integrated clinical response to endometriosis in Kenya that includes psychological support as well as pain management [47]. 

Despite their issues within the medical system, including financial and geographic barriers to accessing care, most participants advised other adolescent girls and women to seek medical care as soon as they noticed unusual symptoms. This highlights the importance of improving local access to preventative reproductive healthcare. Qualitative evidence about barriers to cervical cancer screening and maternity care in Kenya suggests that cultural insensitivity from formal healthcare providers, as well as financial barriers to transportation and childcare, may discourage women from seeking preventative care [48,49]. While Kenya has a menstrual health and hygiene policy [50], integrating these principles across all levels of care is a challenge in the context of limited infrastructural and provider resources. The policy also includes little mention of menstrual disorders such as endometriosis, highlighting the need for more awareness and policy action. Further, reproductive health conditions such as endometriosis may also receive less attention and resources due to the continued high prevalence of other sexual and reproductive health issues, such as HIV and AIDS or adolescent pregnancy [25]. 

Finally, noting the many system and societal challenges involved in seeking treatment for endometriosis, participants advised other women experiencing symptoms to develop strong self-efficacy. Recent research from the United Kingdom has found that self-efficacy is associated with both the mental and physical quality of life among endometriosis patients [51], and a study in Australia identified similar themes wherein women “took control” of their endometriosis journeys by asserting themselves with medical professionals [39]. Indeed, among our participants, self-efficacy included the ability to advocate for oneself within the constraints of the medical system. In addition, women encouraged others to seek out social support by joining dedicated endometriosis support groups. There is minimal recent evidence on social support among women with endometriosis [52], but our findings suggest that this would be a fruitful avenue for future research as support groups may serve as valuable sources of information and safe spaces for processing challenging emotions and experiences.

There are three limitations to note. One, the study was limited to those women able to respond online during the COVID-19 pandemic and thus will not have captured those women unable to utilize their phones or computers to submit stories. Two, the participants were limited to those participating in a support group for endometriosis, which might inherently contribute to a more positive or well-supported experience. It would be useful to conduct a similar study with those who do not have access to other women experiencing similar realities. Three, the women were primarily, even if not entirely, drawn from those living in an urban center in Kenya. The lived experiences of those in rural Kenya, likely with less access to care and less awareness of endometriosis, are also important to capture.

## 5. Conclusions

Written narratives from Kenyan women revealed the wide-ranging effects of debilitating symptoms and menstrual stigma on women’s daily lives and the complexities of acquiring a diagnosis given health system barriers. Going forward, there is a clear need for improved social awareness of endometriosis and the establishment of clear, effective, and supportive pathways with trained, geographically, and financially accessible healthcare providers for endometriosis diagnosis and treatment. Although Kenyan women wrote about the importance of cultivating self-efficacy and social support as effective ways of coping with a chronic and complex disease, it is essential that more integrated health system services are available for endometriosis, including referrals for mental health treatment and support groups, and that education about menstrual disorders begins in adolescence. This study underscores the importance of capturing the perspectives and recommendations of those living with endometriosis in efforts to improve their health and the quality of their lives. 

## Figures and Tables

**Table 1:** Participant information.

		Range	Average
Age		22–48	32.2
		*n*	Percentage
Marital status	single	20	53%
	married	14	37%
	other	2	5%
	divorced	1	3%
Children	yes	9	24%
	no	28	74%
Residence	urban	33	84%
	rural	4	11%

## Data Availability

The data presented in this study may be available on request from the corresponding author. The data are not publicly available due to privacy restrictions.
